# Supplementary material for: Angiotensin II mediates hypertensive cardiac fibrosis via an Erbb4-IR-dependent mechanism
Source: Mol Ther Nucleic Acids. 2023 Jun 26;33:180–90. doi: 10.1016/j.omtn.2023.06.017 (PMC10336735; doi:10.1016/j.omtn.2023.06.017)
Supplement: Document S1. Figure S1 and Table S1 [file mmc1.pdf]

OMTN, Volume *33*

Supplemental information

Angiotensin II mediates hypertensive cardiac fibrosis via an Erbb4-IR-dependent mechanism

Jian-Chun Li, Jian Jia, Li Dong, Zhong-Jing Hu, Xiao-Ru Huang, Hong-Lian Wang, Li Wang, Si-Jin Yang, and Hui-Yao Lan

Table S1. List of PCR primers.

Primers	Forward 5'-3'	Reverse 5'-3'
Erbb4-IR	AACTCGCCACAGAAA TCCAC	ACAACCCCAAACAAGCTGTC
GAPDH	TGCTGAGTATGTCGT GGAGTCTA	AGTGGGAGTTGCTGTTGAAA TC
TGF-β1	CAACAATTCCTGGCG TTACCTTGG	GAAAGCCCTGTATTCCGTCT CCTT
collagen I	GAGCGGAGAGTACTG GATCG	TACTCGAACGGGAATCCATC
collagen III	CTGGACCAAAAGGTG ATGCTG	TGCCAGGGAATCCTCGATGT C
α-SMA	ACTGGGACGACATGG AAAAG	CATCTCCAGAGTCCAGCACA
fibronectin	ACACGGTTTCCCATTA CGCCAT	AATGACCACTGCCAAAGCC CAA
Smad7	GTGCTATGTCGCTCT GGACTTTGA	ATGAAAGATGGCTGGAAGA GGGTC
mCherry	TTCATGTACGGCTCCA AGGC	TGTAGATGAACTCGCCGTCC
miR-29b	ATCTTTGTATCTAGCA CCATT	AATGGTGCTAGATACAAAGA T
U6(universal)	GTGCTCGCTTCGGCAGCACATATACTAAAATTGGAACGA TACAGAGAAGATTAGCATGGCCCCTGCGCAAGGATGACA CGCAAATTCGTGAAGCGTTCCATATTTT	

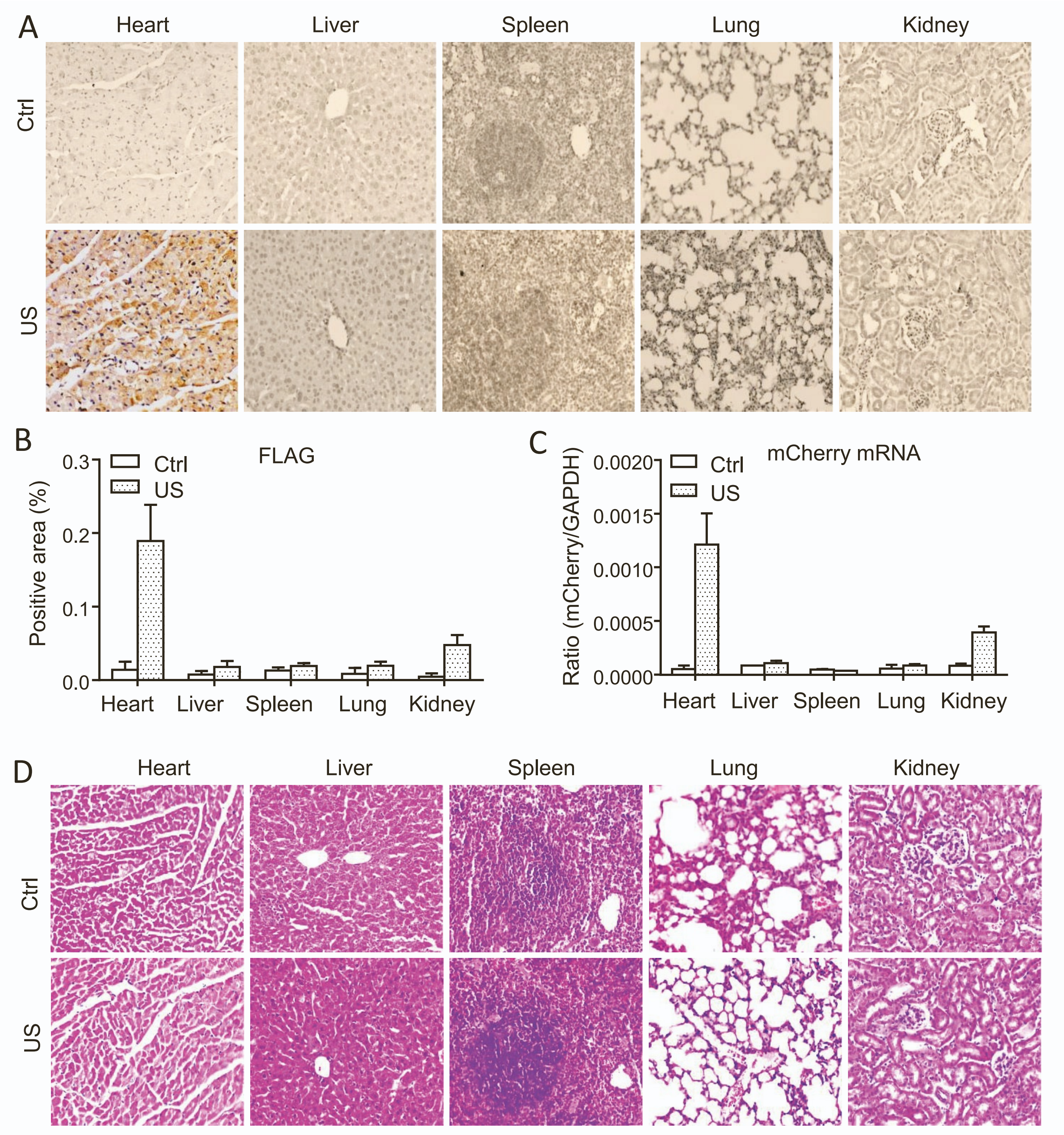

Figure S1. Ultrasound-microbubble mediated cardiac-specific FLAG-mCherry (reporter gene) expression without detectable organ histological injury. (**A,B**) Immunohistochemistry and quantitative analysis for FLAG-mCherry protein expression. (**C**) Real-time PCR for mCherry mRNA expression. (**D**) PAS-staining. Data represent as the mean±SD for groups of 3 mice.
