# Supplementary material for: Angiotensin II mediates hypertensive cardiac fibrosis via an Erbb4-IR-dependent mechanism
Source: Mol Ther Nucleic Acids. 2023 Jun 26;33:180–90. doi: 10.1016/j.omtn.2023.06.017 (PMC10336735; doi:10.1016/j.omtn.2023.06.017)
Supplement: Document S2. Article plus supplemental information [file mmc2.pdf]

Molecular Therapy
Nucleic Acids
Original Article

Angiotensin II mediates hypertensive cardiac fibrosis via an Erbb4-IR-dependent mechanism

Jian-Chun Li,[1,2,5] Jian Jia,[1,5] Li Dong,[2,3,5] Zhong-Jing Hu,[2,3] Xiao-Ru Huang,[2,4] Hong-Lian Wang,[1,2,5] Li Wang,[1] Si-Jin Yang,[3] and Hui-Yao Lan[1,2,4]

[1]Research Center of Integrated Traditional Chinese and Western Medicine, The TCM Affiliated Hospital of Southwest Medical University, Luzhou, Sichuan, China; [2]Department of Medicine and Therapeutics, Li Ka Shing Institute of Health Sciences, and Lui Che Woo Institute of Innovative Medicine, The Chinese University of Hong Kong, Hong Kong, China; [3]National Traditional Chinese Medicine Clinical Research Base, The TCM Affiliated Hospital of Southwest Medical University, Luzhou, Sichuan, China; [4]Guangdong-Hong Kong Joint Laboratory on Immunological and Genetic Kidney Diseases, Guangdong Provincial People's Hospital, Guangdong Academy of Medical Sciences, Guangzhou, Guangdong 510080, China

Transforming growth factor β (TGF-β)/Smad3 plays a vital role in hypertensive cardiac fibrosis. The long non-coding RNA (lncRNA) Erbb4-IR is a novel Smad3-dependent lncRNA that mediates kidney fibrosis. However, the role of Erbb4-IR in hypertensive heart disease remains unexplored and was investigated in the present study by ultrasound-microbubble-mediated silencing of cardiac *Erbb4-IR* in hypertensive mice induced by angiotensin II. We found that chronic angiotensin II infusion induced hypertension and upregulated cardiac *Erbb4-IR*, which was associated with cardiac dysfunction, including a decrease in left ventricle ejection fraction (LVEF) and LV fractional shortening (LVFS) and an increase in LV mass. Knockdown of cardiac *Erbb4-IR* by *Erbb4-IR* short hairpin RNA (shRNA) gene transfer effectively improved the angiotensin II-induced deterioration of cardiac function, although blood pressure was not altered. Furthermore, silencing cardiac *Erbb4-IR* also inhibited angiotensin II-induced progressive cardiac fibrosis, as evidenced by reduced collagen I and III, alpha-smooth muscle actin (α-SMA), and fibronectin accumulation. Mechanistically, improved hypertensive cardiac injury by specifically silencing cardiac *Erbb4-IR* was associated with increased myocardial *Smad7* and *miR-29b*, revealing that *Erbb4-IR* may target *Smad7* and *miR-29b* to mediate angiotensin II-induced hypertensive cardiac fibrosis. In conclusion, *Erbb4-IR* is pathogenic in angiotensin II (Ang II)-induced cardiac remodeling, and targeting *Erbb4-IR* may be a novel therapy for hypertensive cardiovascular diseases.

INTRODUCTION

Hypertensive heart disease is a leading cause of cardiovascular morbidity and mortality.[1] Left ventricle (LV) damage, hypertrophy, and fibrosis are the hallmarks of hypertensive cardiac remodeling, which eventually induces chronic heart failure.[2,3] Hypertensive cardiac remodeling is influenced by a multitude of factors, including the renin-angiotensin-aldosterone system (RAAS) and transforming growth factor β1 (TGF-β1). TGF-β is a key mediator in tissue fibrosis in chronic cardiac and renal diseases.[4,5] Notably, angiotensin II (Ang II) is a key effector molecule of the RAAS and can promote cardiac inflammation and fibrosis via TGF-β-dependent and -independent mechanisms.[6,7] Under hypertensive conditions, Smad3 can be activated directly by the Ang II-TGF-β axis and the Ang II type 1 receptor (AT1)-ERK (extracellular signal-regulated kinase)/p38 MAPK (mitogen-activated protein kinase) crosstalk pathway.[6–9] Thus, targeting Smad3 via genetic deletion or pharmacological inhibition of Smad3 can inhibit hypertensive cardiopathy and nephropathy.[10–12] However, Smad3 deficiency may also trigger autoimmune disease by impairing immunity.[13] This suggests that inhibition of Smad3-dependent molecules specifically associated with cardiac fibrosis, rather than targeting the entire TGF-β/Smad3 signaling, may be a better approach to treat hypertensive cardiovascular disease.

Non-coding RNAs (ncRNAs) have been shown to play a critical role in the pathogenesis of cardiac remodeling.[14–18] By using the RNA sequencing technique, we have previously identified a series of Smad3-dependent long ncRNAs (lncRNAs) related to renal fibrosis and inflammation in rodent models.[19,20] Of them, the lncRNA *Erbb4-IR* is a novel Smad3-dependent lncRNA that mediates renal

Received 5 January 2023; accepted 22 June 2023;
https://doi.org/10.1016/j.omtn.2023.06.017.

[5]These authors contributed equally

Correspondence: Li Wang, Research Center of Integrated Traditional Chinese and Western Medicine, The TCM Affiliated Hospital of Southwest Medical University, Luzhou, Sichuan, China.
E-mail: wangli120@swmu.edu.cn
Correspondence: Si-Jin Yang, Department of Cardiovascular Medicine, The TCM Affiliated Hospital of Southwest Medical University, 182 Chunhui Road, Luzhou, Sichuan 646000, China.
E-mail: ysjimn@swmu.edu.cn
Correspondence: Hui-Yao Lan, Department of Medicine and Therapeutics, Li Ka Shing Institute of Health Sciences, The Chinese University of Hong Kong, Hong Kong, China.
E-mail: hylan@cuhk.edu.hk

 © 2023 The Author(s).
This is an open access article under the CC BY license (http://creativecommons.org/licenses/by/4.0/).

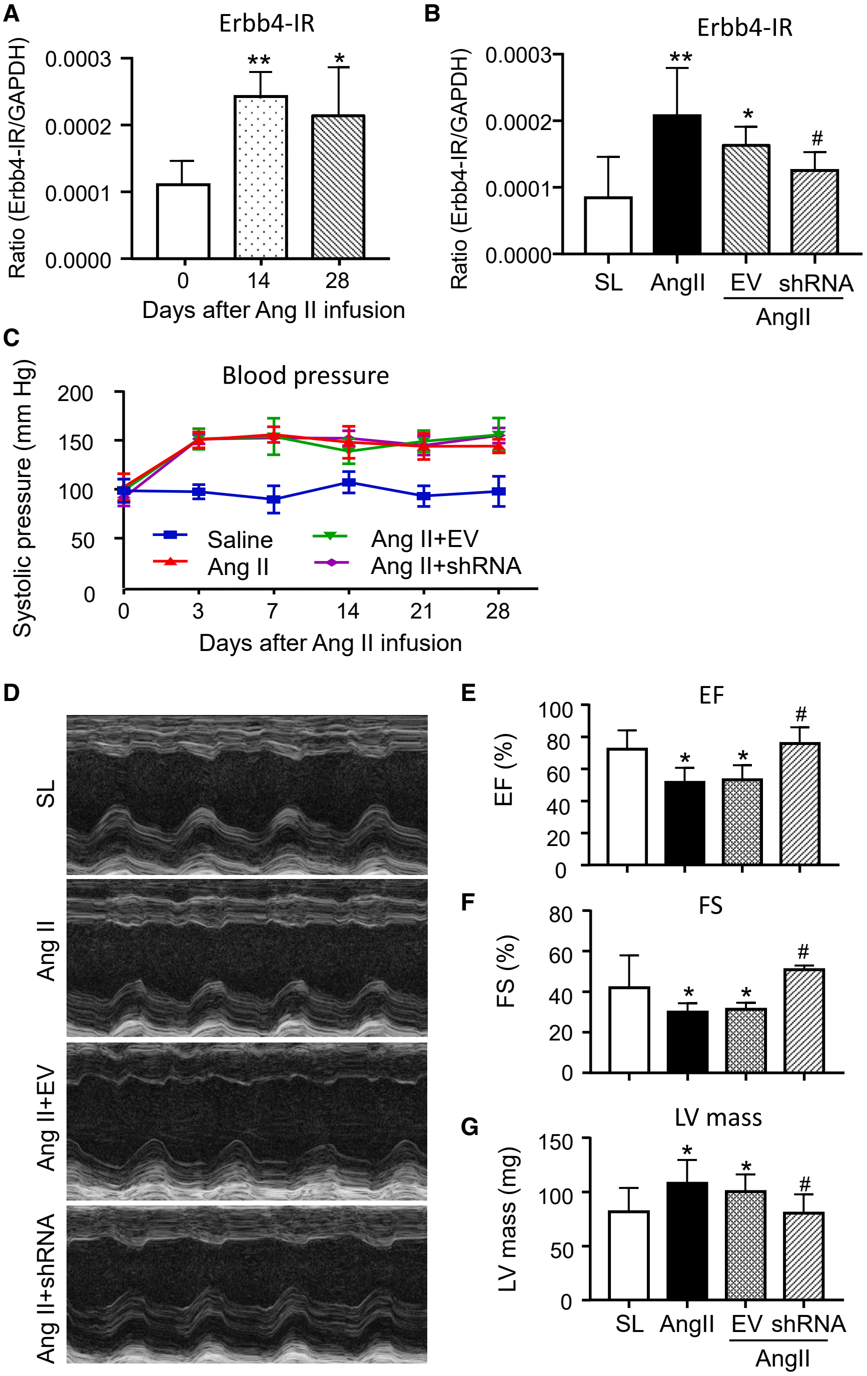

Figure 1. Cardiac *Erbb4-IR* is highly upregulated in the hypertensive heart, and silencing cardiac *Erbb4-IR* protects against cardiac dysfunction in Ang II-induced hypertensive mice

(A) Real-time PCR detects the expression of *Erbb4-IR* in the LV tissue of mice with or without Ang II infusion (1.46 mg/kg/day) on days 0, 14, and 28. (B) Expression of cardiac *Erbb4-IR* in mice treated with or without Ang II and ultrasound-microbubble-mediated *Erbb4-IR* shRNA-expressing plasmid. (C) Levels of systolic blood pressure. (D) Representative echocardiographic images (M mode). (E–G) Echocardiography assessments of LV ejection fraction (LVEF), LV fractional shortening (LVFS), and LV mass index. Data are presented as mean ± SEM for a group of eight mice. *p < 0.05, **p < 0.01 compared with the saline group (SL); ##p < 0.01 compared with Ang II + empty vector (EV) control.

fibrosis in obstructive and diabetic nephropathy.[21,22] We found that *Erbb4-IR* is tightly regulated by Smad3 in response to TGF-β1 and advanced glycation end products.[21,22] We also find that specifically targeting *Erbb4-IR* can inhibit renal fibrosis in mouse models of obstructive and diabetic nephropathy.[21,22] However, it is unclear whether Erbb4-IR plays a pathogenic role and whether it could be a therapeutic target for hypertensive heart disease. Thus, this study aims to examine the role of *Erbb4-IR* for hypertensive heart disease by specifically silencing cardiac *Erbb4-IR* in a mouse model of Ang II-induced hypertension to determine its potential as a therapeutic target.

RESULTS

Silencing cardiac *Erbb4-IR* inhibits cardiac dysfunction

Because expression of lncRNAs is often at a low level and in a tissue-dependent manner, we first evaluated the expression level of cardiac *Erbb4-IR* in response to Ang II infusion. Real-time PCR detected that Ang II infusion for 14 consecutive days resulted in a significant increase in *Erbb4-IR* in the LV, which remained high on day 28 (Figure 1A) but was largely inhibited by overexpression of *Erbb4-IR* shRNA (Figure 1B). Next, we determined the pathogenic role and therapeutic effect of Erbb4-IR on Ang II-induced hypertensive heart disease by ultrasound-microbubble-mediated overexpression of *Erbb4-IR* shRNA. As shown in Figures 1C–1G, Ang II infusion resulted in hypertension and cardiac dysfunction, with a significant increase in systolic blood pressure and a reduction in LV ejection fraction (LVEF) and LV fractional shortening (LVFS) as well as an increase in LV mass when compared with saline-treated mice. However, there was no difference in Ang II-induced hypertension and cardiac dysfunction between groups of mice that received Ang II with or without control empty vector (EV) treatment. Interestingly, compared with the EV-treated animals, treatment with *Erbb4-IR* shRNA did not alter the elevated levels of blood pressure induced by Ang II (Figure 1C) but largely inhibited Ang II-induced cardiac dysfunction by significantly increasing LVEF and LVFS while reducing LV mass (Figures 1D–1G).

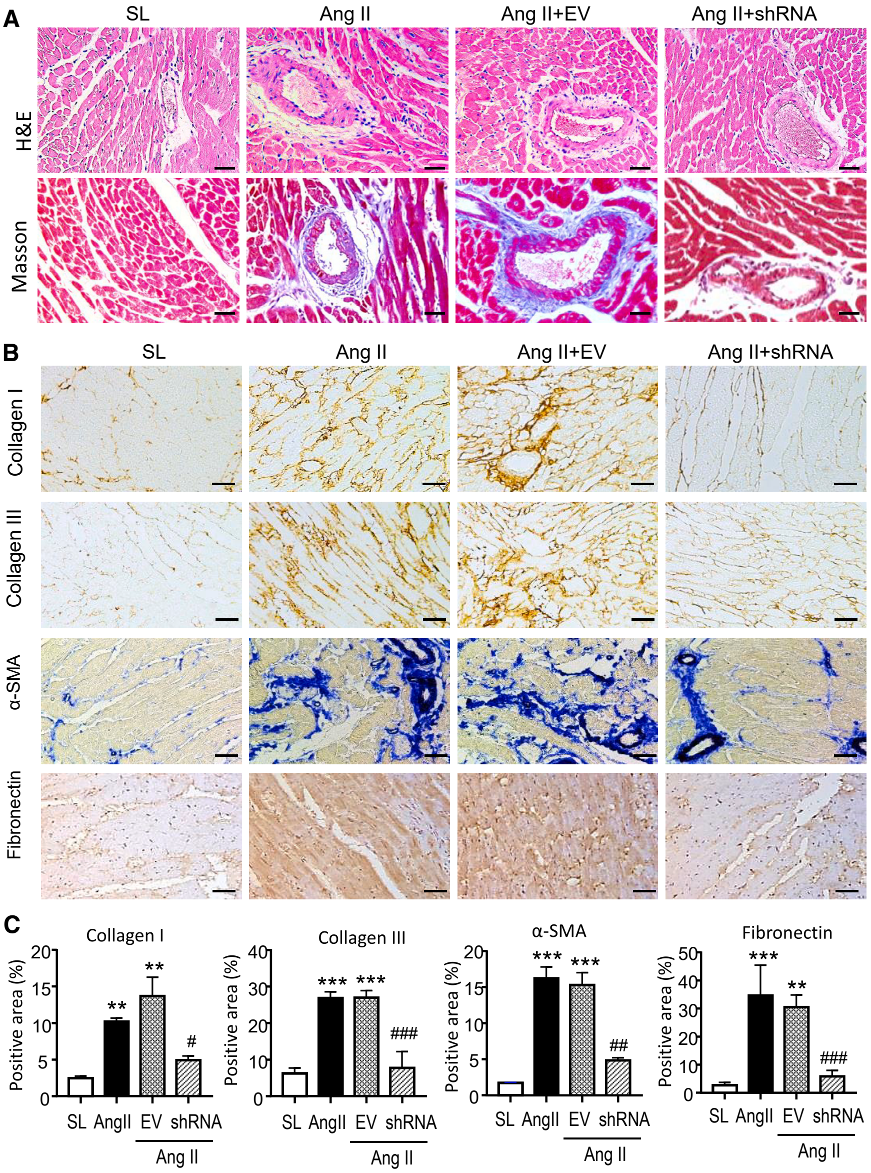

Figure 2. Silencing cardiac *Erbb4-IR* prevents Ang II-induced cardiac fibrosis in hypertensive mice

(A) Representative images of H&E and Masson's trichrome staining. (B) Representative immunohistochemical images of collagen I, collagen III, α-SMA, and fibronectin. (C) Quantitative analysis of collagen I, collagen III, α-SMA, and fibronectin from immunohistochemical staining. Data are presented as mean ± SEM for a group of eight mice. **p < 0.01, ***p < 0.001 compared with SL; #p < 0.05, ##p < 0.01 compared with Ang II + EV control. Scale bars, 20 μm.

Silencing cardiac *Erbb4-IR* effectively attenuates myocardial fibrosis in Ang II-induced hypertensive mice

Cardiac fibrosis is the hallmark leading to LV dysfunction and heart failure. Hence, we further investigated the protective effect of silencing cardiac *Erbb4-IR* on Ang II-induced hypertensive myocardial remodeling. H&E and Masson's trichrome staining revealed moderate myocardial fibrosis, identified by extracellular matrix deposition in Ang II-induced hypertensive mice, which was abrogated in those treated with *Erbb4-IR* shRNA but not by the EV control (Figure 2A). Immunohistochemistry confirmed aberrant deposition of collagen I, collagen III, α-SMA, and fibronectin in the LV tissues of Ang II infusion mice treated with or without EV (Figures 2B and 2C). In contrast, treatment with *Erbb4-IR* shRNA significantly blunted Ang II-induced cardiac fibrosis by greatly inhibiting collagen I and III, α-SMA, and fibronectin accumulation in the LV tissues (Figures 2B and 2C). This was further demonstrated at the total protein levels by western blotting, where Ang II-induced marked accumulation of collagen I and III, α-SMA, and fibronectin in the LV tissues was almost completely blocked by treatment with *Erbb4-IR* shRNA but not by the EV control treatment (Figure 3). Further study by real-time PCR also confirmed the notion that silencing cardiac *Erbb4-IR* protected against Ang II-induced upregulation of *collagen I* and *III, α-SMA*, and *fibronectin* at the mRNA levels (Figure 4).

Restored expression of cardiac *Smad7* and *miR-29b* is a mechanism through which silencing *Erbb4-IR* inhibits Ang II-induced cardiac fibrosis

We next investigated the mechanism by which silencing cardiac *Erbb4-IR* ameliorated Ang II-induced hypertensive cardiac fibrosis. In our previous studies, we identified that *Smad7* and *miR-29b* (*miR-29b-3p*) are the direct target genes of *Erbb4-IR* and *Erbb4-IR* functions as an integrated effector molecule in the positive feedback circuit of TGF-β/Smad signaling to inhibit *Smad7* and *miR-29b* in obstructive nephropathy and diabetic nephropathy.[21,22] We then investigated whether silencing cardiac *Erbb4-IR* inhibits Ang II-induced hypertensive cardiac fibrosis by upregulating cardiac *Smad7* and *miR-29b*. The results shown in Figure 5 demonstrated that Ang II infusion caused strong activation of TGF-β/Smad3 signaling, as identified by marked expression of *TGF-β1*, phosphorylation of Smad2/3, and nuclear translocation of phosphorylated Smad2/3 in LV tissues, which was associated with a loss of cardiac *Smad7* and *miR-29* (Figure 6). Treatment with ultrasound-mediated *Erbb4-IR* shRNA-expressing plasmids was associated with inhibition of Ang II-induced *Erbb4-IR* (Figure 1A) and an increase in cardiac *Smad7* and *miR-29* expression (Figure 6), implying that *Erbb4-IR* may mediate Ang II-induced cardiac fibrosis by downregulating cardiac *Smad7* and *miR-29b*.

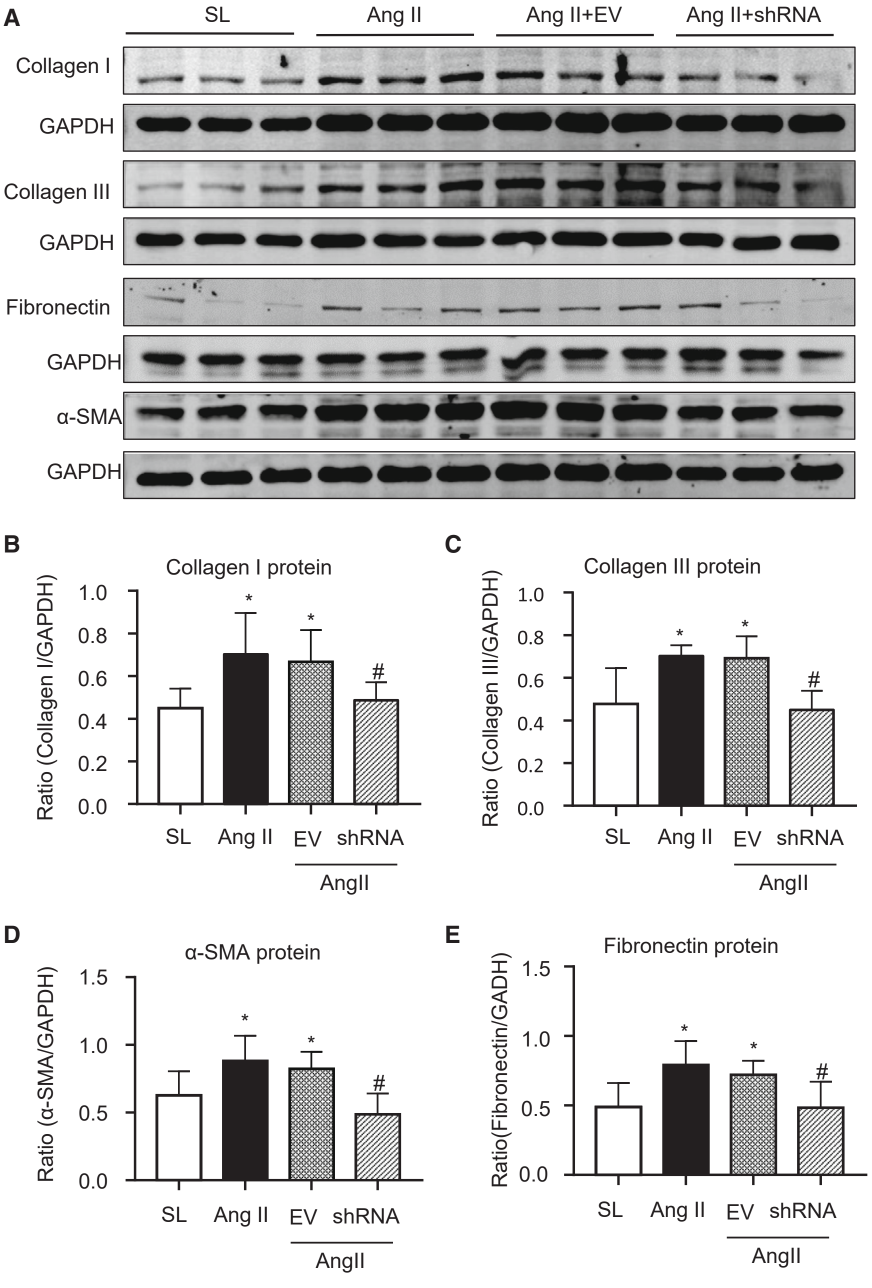

Figure 3. Western blot analysis detects that silencing cardiac *Erbb4-IR* inhibits cardiac fibrosis in Ang II-induced hypertensive mice

(A) Representative western blots for collagen I, collagen III, α-SMA, and fibronectin from the LV tissues of mice treated with or without Ang II and *Erbb4-IR* shRNA-expressing plasmids. (B–E) Quantitative analysis of collagen I, collagen III, α-SMA, and fibronectin. Data are presented as mean ± SEM for a group of eight mice. *p < 0.05 compared with SL; #p < 0.05 compared with Ang II + EV control.

Silencing Erbb4-IR inhibits in Ang II-induced cardiac fibrosis *in vitro*

To further investigate the regulatory role and mechanisms of *Erbb4-IR* in cardiac fibrosis induced by Ang II, we examined Ang II-induced Erbb4-IR expression in isolated cardiac fibroblasts (CFs) and cardiomyocytes from C57BL/6 neonatal mouse hearts. The results shown in Figures 7A and 7B revealed that addition of Ang II significantly increased *Erbb4-IR* expression in CFs but not in cardiomyocytes in a time-dependent manner, suggesting a regulatory role of Erbb4-IR in CF-mediated cardiac fibrosis. Importantly, real-time PCR and western blot analysis also showed that silencing *Erbb4-IR* significantly blocked Ang II-induced cardiac fibrosis by inhibiting expression of collagen I, III, α-SMA, and fibronectin (Figures 7C–7L). Mechanistically, we uncovered that silencing *Erbb4-IR* in CFs restored the balance of TGF-β/Smad signaling by inhibiting Ang II-induced Smad2/3 phosphorylation while increasing Smad7 expression (Figures 8A and 8B). Furthermore, real-time PCR also detected that silencing Erbb4-IR protected against Ang II-induced loss of miR-29b in CFs (Figure 8C).

DISCUSSION

Increasing evidence has demonstrated that Ang II is a key mediator in hypertensive cardiac disease. In this study, we identified that Ang II mediates hypertensive cardiac disease via an lncRNA Erbb4-IR-dependent mechanism. Indeed, chronic Ang II infusion markedly upregulated cardiac *Erbb4-IR*, which was associated with hypertension and development of hypertensive cardiac disease, as demonstrated by significant reductions in LVEF and LVFS and increases in LV mass and cardiac fibrosis. To confirm the pathogenic role of Erbb4-IR in the pathogenesis of hypertensive heart disease, we specifically knocked down cardiac *Erbb4-IR* by ultrasound-mediated *Erbb4-IR* shRNA-expressing plasmids and found that targeting cardiac *Erbb4-IR* blocked Ang II-induced hypertensive cardiac disease by increasing the LVEF and LVFS while reducing LV mass and inhibiting cardiac fibrosis. The pathogenic role of Erbb4-IR in cardiac fibrosis was further confirmed *in vitro*, where addition of Ang II was capable of inducing expression of Erbb4-IR in CFs but not in cardiomyocytes, revealing that Erbb4-IR is expressed by fibrogenic cells in response to Ang II in the heart. Again, specifically silencing *Erbb4-IR* also inhibited Ang II-induced collagen matrix and α-SMA expression by CFs. The restensive cardiopathy via an Erbb4-IR-dependent mechanism and that targeting *Erbb4-IR* may be a promising and effective therapeutic strategy for hypertensive cardiac disease.

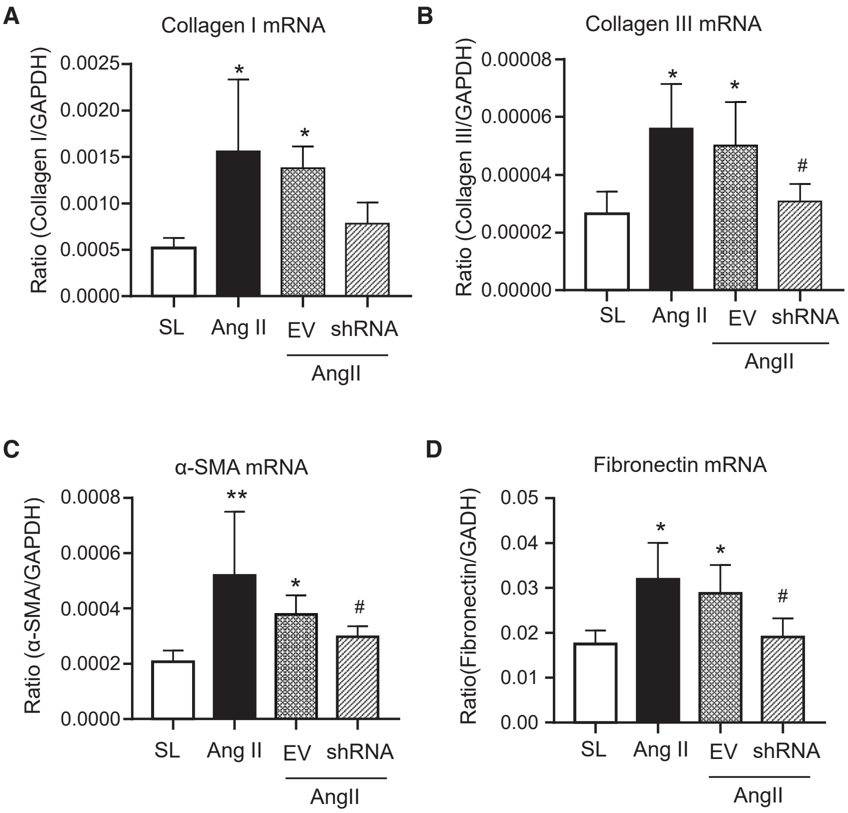

Figure 4. Real-time PCR shows that silencing cardiac *Erbb4-IR* inhibits cardiac fibrosis in Ang II-induced hypertensive heart disease

(A) *Collagen I* mRNA. (B) *Collagen III* mRNA. (C) *α-SMA* mRNA. (D) *Fibronectin* mRNA expression. Data are the mean ± SEM for a group of eight mice. *$p < 0.05$, **$p < 0.01$ compared with SL; #$p < 0.05$ compared with Ang II + EV control.

It is possible that lncRNA *Erbb4-IR* may mediate Ang II-induced cardiac disease by reducing cardiac *Smad7*. This is consistent with our previous finding that *Erbb4-IR* acts as an integrated effector molecular to promote renal fibrosis by targeting *Smad7* by directly binding to the 3′ UTR of the *Smad7* genomic sequence.[21] It has been well established that Ang II induces hypertensive cardiopathy and nephropathy by activating TGF-β/Smad3 signaling directly and indirectly via the TGF-β and ERK/p38 MAPK-Smad crosstalk pathways.[6–12] Among TGF-β/Smad signaling, Smad3 is pathogenic because genetic deletion or pharmacological inhibition of Smad3 can protect against Ang II-induced hypertensive cardiopathy and nephropathy.[10,11] In contrast, Smad2 is protective in terms of tissue fibrosis.[23] Therefore, Smad3 and Smad2 play a distinct role in cardiac fibrosis in the infarcted heart.[24–26] In contrast, Smad7 is heart protective because Smad7 deficiency promotes but overexpression of cardiac Smad7 inhibits Ang II-induced cardiac fibrosis.[27,28] Our recent study found that Smad7 is a target gene of Erbb4-IR because a binding site of Erbb4-IR is found on the 3′ UTR of the *Smad7* gene, and mutation of this binding site can prevent the suppressive effect of Erbb4-IR on *Smad7* reporter activity.[21] Thus, Ang II may signal via Smad3 to induce overexpression of cardiac *Erbb4-IR* to mediate cardiac fibrosis by downregulating cardiac *Smad7*, as seen in the present study *in vivo* and *in vitro*. Interestingly, we also found that inhibition of cardiac *Erbb4-IR* reduced the levels of p-Smad3 while increasing Smad7 in the hypertensive heart and in Ang II-stimulated CFs, indicating a Smad3-Erbb4-IR circuit mechanism during Ang II-induced cardiac fibrosis *in vivo* and *in vitro*. Although silencing *Erbb4-IR* also inhibited p-Smad2, our findings suggest that the Smad3-Erbb4-IR circuit plays a key role in Ang II-induced cardiac fibrosis, as evidenced by the suppressive effect of Erbb4-IR silencing on cardiac fibrosis. Thus, restoring the balance of TGF-β/Smad signaling by inhibiting Smad3 while increasing Smad7 may be a mechanism by which targeting Erbb4-IR inhibits Ang II-induced cardiac fibrosis.

Additionally, Erbb4-IR may also mediate hypertensive cardiac fibrosis by reducing cardiac *miR-29b* expression. It is well recognized that *miR-29* is a key regulator in the pathogenesis of cardiac fibrosis.[29,30] We have also reported that *miR-29b* is negatively regulated by Smad3 and that overexpression of *miR-29b* is able to inhibit Ang II-mediated hypertensive cardiac fibrosis as well as obstructive kidney and lung fibrosis.[31–33] Importantly, our recent study also found that the Erbb4-IR-miR-29b axis is a key mechanism of type 2 diabetic nephropathy because *Erbb4-IR* can bind the 3′ UTR of the *miR-29b* genomic sequence to suppress *miR-29b* expression at the transcriptional level.[22] Thus, inhibition of *Erbb4-IR* can protect against diabetic nephropathy by upregulating renal *miR-29b*.[22] In line with this notion, the present study also discovered that chronic Ang II infusion markedly upregulated cardiac *Erbb4-IR* while reducing cardiac *miR-29b* expression in the hypertensive heart. Conversely, silencing cardiac *Erbb4-IR* increased the levels of cardiac *miR-29b*, suggesting that this pathway contributes to inhibition of cardiac fibrosis, as observed in the present study. *In vitro* studies in CFs also confirmed the notion that silencing Erbb4-IR in CFs blocked Ang II-induced loss of *miR-29b* and cardiac fibrosis. Thus, up-regulation of *miR-29b* may be an additional mechanism by which targeting *Erbb4-IR* protects against Ang II-mediated cardiac fibrosis.

In the present study, we specifically knocked down cardiac *Erbb4-IR* by ultrasound-microbubble-mediated *Erbb4-IR* shRNA overexpression. This technique is based on using ultrasound waves to destroy the gene-bearing microbubbles and release the vectors locally to the target tissues. We used specific ultrasound parameters (2 W/cm^2) and microbubble doses to ensure that the microbubbles mainly burst in the heart when they are circulating within the heart tissues where ultrasound

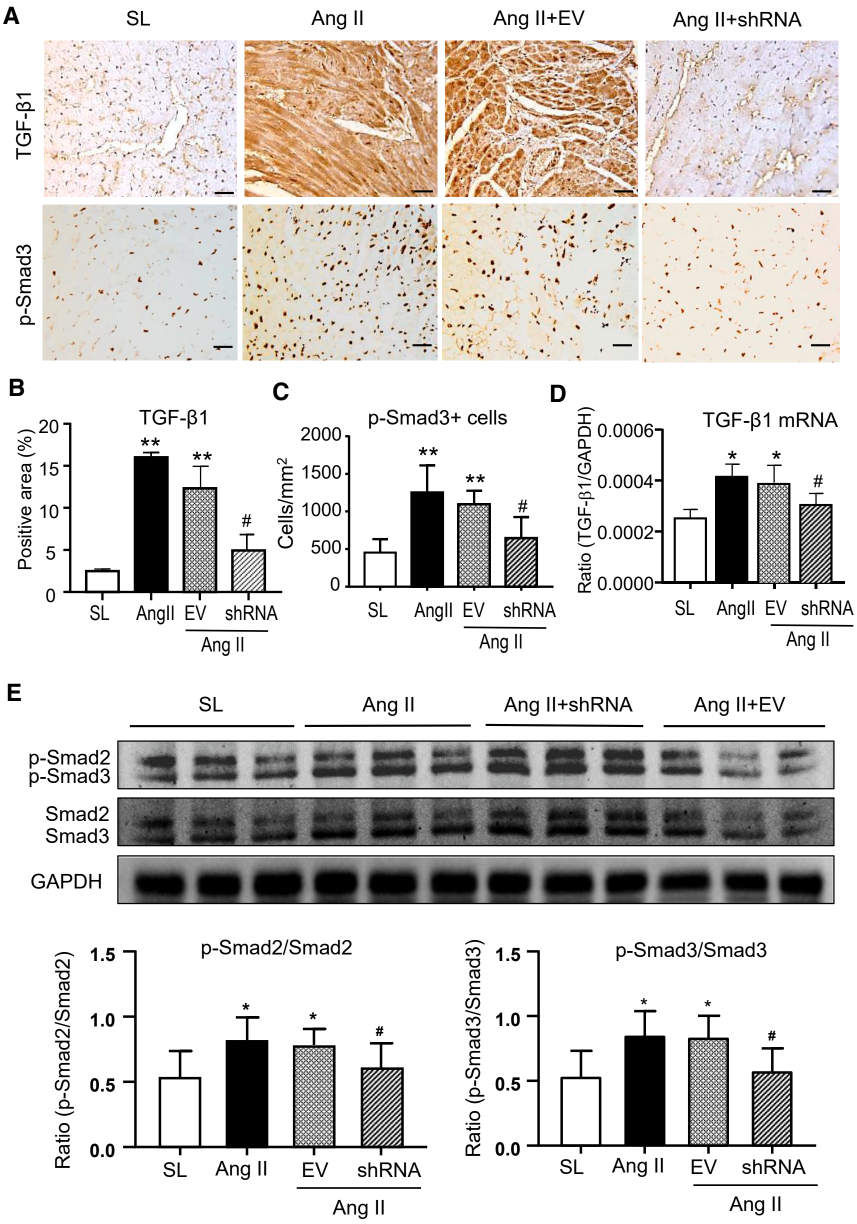

Figure 5. Knockdown of cardiac *Erbb4-IR* blocks activation of myocardial TGF-β/Smad3 signaling in Ang II-induced hypertensive mice

(A) Representative immunohistochemical images of TGF-β1 and p-Smad3 in the LV tissues of mice treated with or without Ang II and *Erbb4-IR* shRNA-expressing plasmids. (B and C) Quantitative analysis of TGF-β1 and p-Smad3 from immunohistochemical staining. (D) Real-time PCR analysis of *TGF-β1* mRNA expression. (E) Western blot and quantitative analysis of p-Smad2 and p-Smad3. Data are the mean ± SEM for a group of eight mice. *p < 0.05, **p < 0.01 compared with SL; #p < 0.05 compared with Ang II + EV control. Scale bars, 20 μm.

waves are exposed. The mechanism of ultrasound-microbubble-mediated gene transfer has been described previously.[34] Briefly, use of ultrasound contrast agents can lower the threshold for cavitation by ultrasound energy. After adhering to the surface of microbubbles, genes or expression vectors, such as *Erbb4-IR* shRNA-expressing plasmids, can be injected intravenously, and ultrasound energy is then applied locally to the target region, such as the heart in this study. As the gene-bearing microbubbles enter the region of insonation, they cavitate, locally releasing the gene, such as the *Erbb4-IR* shRNA-expressing plasmid, into the heart tissue. Cavitation can also increase cell permeability, improving cellular uptake of released DNA. By using FLAG-fused mCherry as a reporter gene, we were able to highly express it in the heart but not in other organs, including the lungs, liver, kidneys, or spleen, indicating a tissue-specific and low off-target effect of ultrasound-microbubble-mediated gene transfer. Furthermore, we found no detectable side effects on histological injury in various tissues, indicating the safety of ultrasound-microbubble-mediated gene therapy.

In summary, the present study identifies the pathogenic role of *Erbb4-IR* in Ang II-mediated cardiac fibrosis. We also uncover that *Erbb4-IR* mediates Ang II-induced cardiac fibrosis by downregulating *Smad7* and *miR-29b*. Thus, targeting *Erbb4-IR* may be a novel and effective therapy for hypertensive cardiovascular disease.

MATERIALS AND METHODS

Ang II-induced hypertensive mouse model

Hypertension was established in mice (C57BL/6 background, male, age of 8–10 weeks) by subcutaneous infusion of Ang II at a dose of 1.46 mg/kg/day for 28 days by implanting an osmotic pump (model 2004; ALZA, Palo Alto, CA, USA) at the dorsum of the mouse back as described previously.[10,11,27,28] Control animals followed the same experimental procedure but received a saline infusion only. In addition, groups of 8 normal mice were used as age-matched controls. All mice were sacrificed on day 28 after Ang II infusion, and the left ventricular tissues were collected for subsequent analysis by western blotting, real-time PCR, and immunohistochemistry on day 28 after Ang II infusion. The experimental protocol was approved by the Animal Experimentation Ethics Committee, Chinese University of Hong Kong (reference numbers 13-057-MIS and 17-178-MIS).

Ultrasound-mediated gene transfer of Erbb4-IR shRNA plasmids into the mouse heart

To investigate the pathogenic role and therapeutic potential of *Erbb4-IR* in Ang II-induced hypertensive cardiac disease, *Erbb4-IR* shRNA-expressing plasmids were delivered into the mouse heart

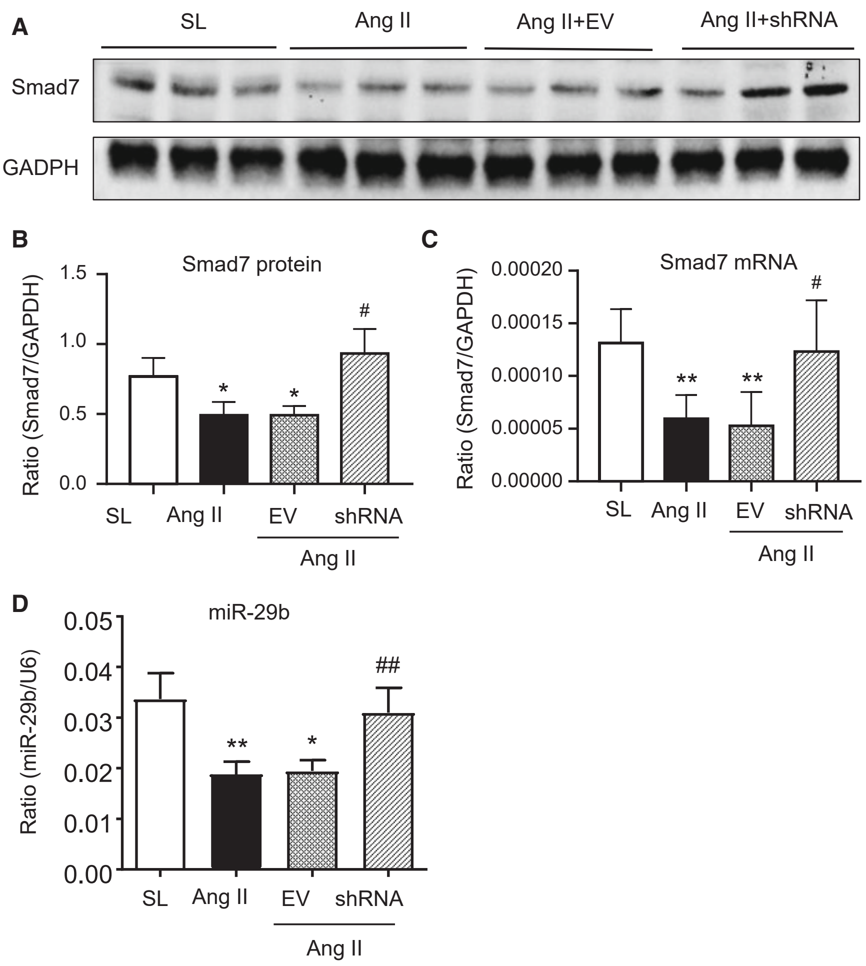

Figure 6. *Erbb4-IR* targets cardiac *Smad7* and *miR-29b* to mediate cardiac fibrosis in Ang II-induced hypertensive mice

(A) Representative western blots of Smad7 protein expression in the LV tissues of mice treated with or without Ang II and *Erbb4-IR* shRNA-expressing plasmids. (B) Quantitative analysis of cardiac Smad7 protein expression. (C) Real-time PCR analysis of cardiac *Smad7* mRNA expression. (D) Real-time PCR analysis of cardiac *miR-29b* expression. Data are the mean ± SEM for a group of eight mice. *p < 0.05, **p < 0.01 compared with SL; #p < 0.05, ##p < 0.01 compared with Ang II + EV control.

at the same time as the Ang II infusion by an ultrasound-microbubble-mediated gene transfer technique as described previously.[28,31] Briefly, after being anesthetized with ketamine (80 mg/kg) and xylazine (15 mg/kg), groups of 8 mice were injected intravenously with a mixture containing either Erbb4-IR shRNA-pSuper.puro vector or empty pSuper.puro vector (200 μg/mouse in 200 μL of saline) and 200 μL of lipid microbubbles (SonoVue, Bracco, Milan, Italy) at a ratio of 1:1 (v/v) via the tail vein over 3–4 min. The microbubbles have an average diameter of 2.5 μm and are composed of a phospholipid shell and a sulfur hexafluoride gas core. The entrapment efficiency of the microbubbles is reported by the manufacturer to be more than 90%. Immediately after injection, non-invasive ultrasound treatment was performed by placing the ultrasound probe (Therasonic, Electro Medical Supplies) on the chest skin over the heart with a plus-wave output at 2 W/cm^2 for a total of 5 min with 30-s intervals. Based on our previous studies, use of 200 μg *Erbb4-IR* shRNA-expressing plasmids can produce a better gene transfection rate and maintain transgene expression for 1–2 weeks.[22] To maintain silencing of *Erbb4-IR* in the mouse heart over the 28-day disease course, the second Erbb4-IR shRNA-expressing plasmid transfer into the hypertensive mouse heart was conducted on day 14. The experimental procedures were approved by the Animal Experimentation Ethics Committee, Chinese University of Hong Kong (reference numbers 13-057-MIS and 17-178-MIS).

To assess the ultrasound-microbubble-mediated gene transfer efficiency into the heart, a reporter gene, FLAG mCherry, was used. Briefly, a mixture of PCDNA3.1-FLAG mCherry (or control vector)-expressing plasmid (200 μg/mouse in 200 μL of saline) and SonoVue (200 μL/mouse) was administered via the tail vein, followed immediately by ultrasound exposure as described above. Groups of three mice were sacrificed daily over the next 2 days to examine FLAG mCherry expression via immunohistochemistry and real-time PCR. The results shown in Figure S1 demonstrated that ultrasound-microbubble-mediated expression of FLAG mCherry was largely detected in the heart, with minimal or undetectable expression in other organs, including the liver, lungs, spleen, and kidneys, without detectable histological injury by H&E staining, confirming a safe, high transfection rate and heart specificity by using the ultrasound-microbubble gene transfer technique.

Blood pressure and echocardiography

Blood pressure was measured in conscious mice before and after Ang II infusion on days 3, 7, 14, and 28 using a noninvasive tail cuff method (CODA High-throughput Non-Invasive Blood Pressure System; Kent Scientific, Torrington, CT, USA) as described previously.[27,28,31] Cardiac functions were examined by echocardiography before and on day 28 after Ang II infusion by transthoracic echocardiography using a Vevo770 high-resolution ultrasound imaging system (VisualSonics, Toronto, ON, Canada) with an RMV 707B scan head (30 MHz; VisualSonics). The mouse's body temperature was maintained at 37°C, and the heart rate was around 450 beats/min. The standard M-mode parameters, including LV mass, LVEF, and LVFS, were calculated according to the guidelines of the Vevo 770 (VisualSonics).

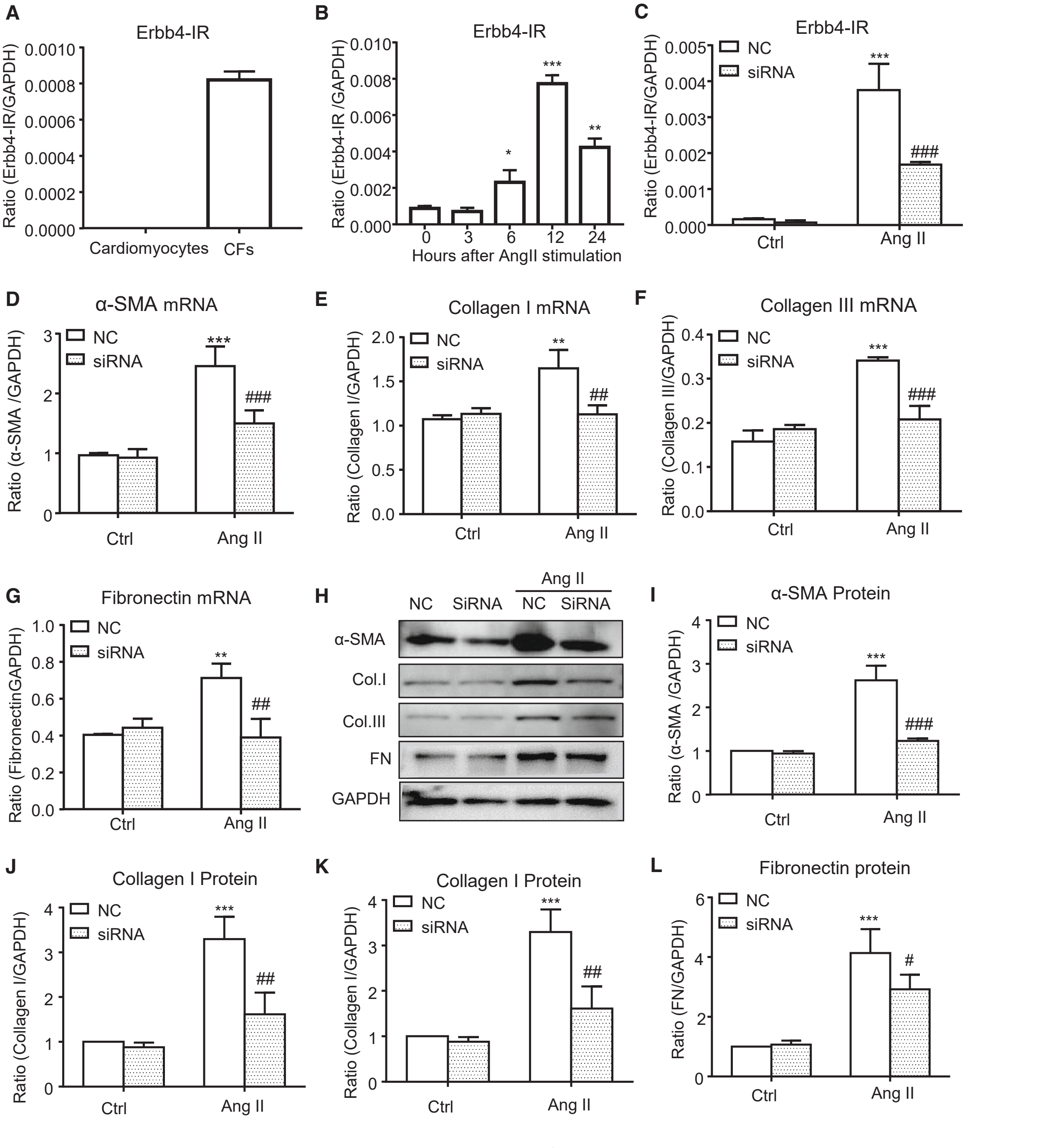

Figure 7. *In vitro* studies detect that addition of Ang II induces *Erbb4-IR* expression by CFs but not by cardiomyocytes and that silencing *Erbb4-IR* protects against Ang II-induced extracellular matrix production by CFs

(A) Addition of Ang II (1 μM) for 12 h markedly upregulates Erbb4-IR in CFs but not in cardiomyocytes. (B) Addition of Ang II (1 μM) upregulates Erbb4-IR in CFs in a time-dependent manner. (C) Expression of Erbb4-IR by CFs treated with or without Ang II (1 μM) and/or *Erbb4-IR* shRNA. (D–G) Real-time PCR reveals that silencing Erbb4-IR inhibits Ang II (1 μM)-induced mRNA expression of α-SMA, collagen I, collagen III, and fibronectin by CFs. (H–L) Western blot analysis shows that silencing Erbb4-IR inhibits Ang II (1 μM)-induced protein expression of α-SMA, collagen I, collagen III, and fibronectin by CFs. Data are the mean ± SEM from three independent experiments. *p < 0.05, **p < 0.01, ***p < 0.001 compared with negative control (Ctrl) without Ang II treatment; #p < 0.05, ##p < 0.01, ###p < 0.001 compared with Ang II + negative EV Ctrl (NC).

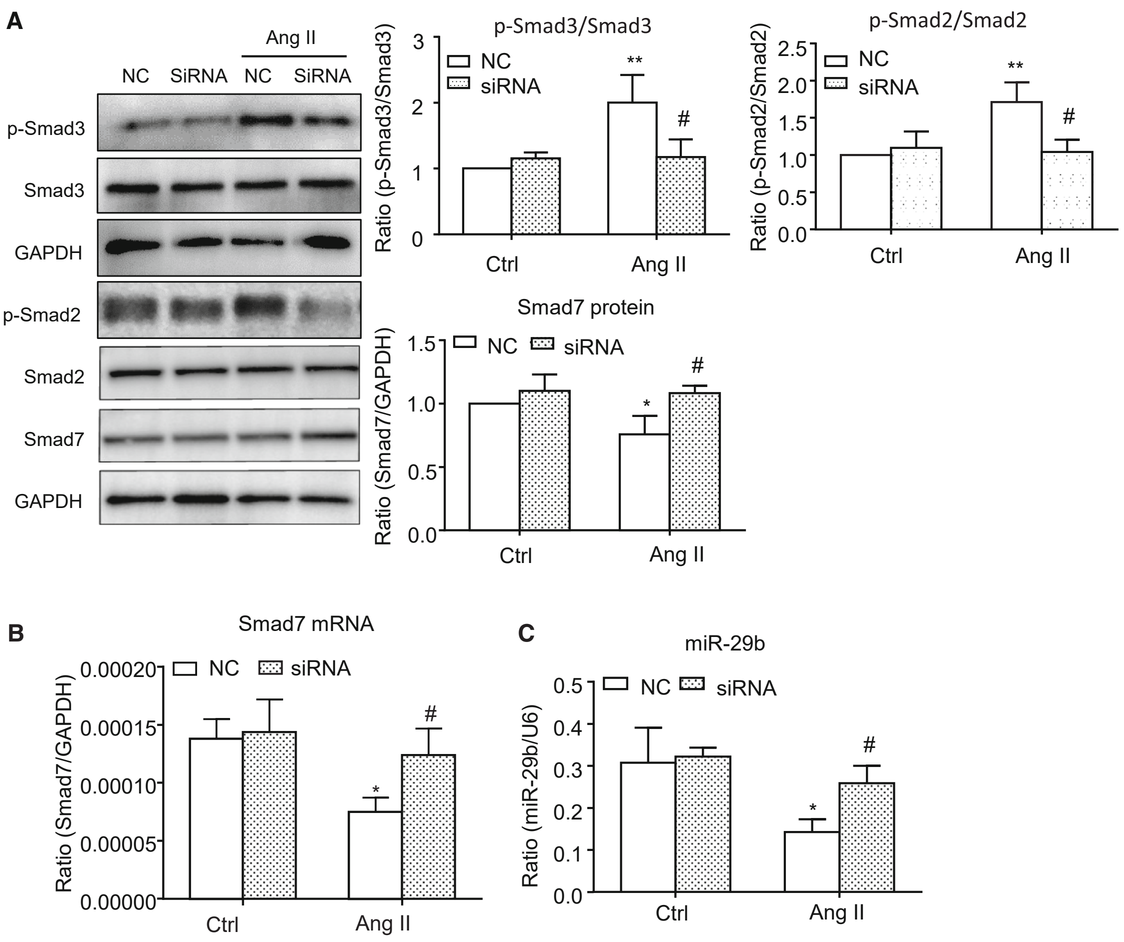

Figure 8. *In vitro* studies detect that silencing *Erbb4-IR* inhibits Ang II-induced Smad2/3 signaling while upregulating Smad7 and miR-29b by CFs

(A) Western blot analysis shows that silencing Erbb4-IR inhibits Ang II (1 μM)-induced p-Smad2 and p-Smad3 while increasing Smad7 in CFs. (B and C) Real-time PCR detects that silencing Erbb4-IR inhibits Ang II (1 μM)-induced loss of Smad7 and miR-29b by CFs. Data are the mean ± SEM from three independent experiments. *$p < 0.05$, **$p < 0.01$ compared with negative Ctrl without Ang II treatment; #$p < 0.05$ compared with Ang II + NC.

Histology and immunohistochemistry

Myocardial injury was determined in paraffin sections (3 μm) from LV mouse heart tissues by hematoxylin and eosin (H&E) and Masson's trichrome staining. In addition, cardiac fibrosis was assessed by immunohistochemistry by means of microwave-based antigen retrieval technique as described previously.[27,28,31] After microwaving, the sections were incubated with primary antibodies against fibronectin and TGF-β1 (catalog numbers sc-8422 and sc-130348, respectively; Santa Cruz Biotechnology, Santa Cruz, CA, USA), phosphorylated Smad3 (p-Smad3) at the pS423 and pS425 residues (catalog number 600-401-919, Rockland, Gilbertsville, PA, USA), collagen I and collagen III (catalog numbers 1310-01 and 1330-01, respectively; Southern Biotech, Birmingham, AL, USA), and α-SMA (catalog number A5228, Sigma, St. Louis, MO, USA) overnight at 4°C. Subsequently, the sections were rinsed with PBS, incubated with the secondary antibody, and visualized with diaminobenzidine. For quantitative analysis of p-Smad3-positive cells, each section was subjected to counting the nucleated p-Smad3-positive cells under a 40× objective field by means of a 0.0625-mm^2 graticule fitted in the eyepiece of the microscope, and nucleated positive cells were expressed as cells per millimeters squared. For expression of TGF-β and accumulation of collagen I and III, fibronectin, and α-SMA, a quantitative image-analysis system (Image-Pro Plus 6.5, Media Cybernetics, Silver Spring, MD, USA) was applied, and positive signals were expressed as a percentage as described previously.[27,28,31]

Western blot analysis

Total protein from the LV was extracted for western blot analysis as described previously.[10,11,24–26] In brief, after blocking nonspecific binding with 5% BSA, the membrane was incubated at 4°C overnight with the indicated primary antibodies, including those against collagen I and III (catalog numbers 1310-01 and 1330-01, respectively; Southern Biotech), α-SMA (catalog number A5228, Sigma), fibronectin (catalog number sc-8422, Santa Cruz Biotechnology), p-Smad2 at the pS465 and pS467 residues/Smad3 at the pS423 and pS425 residues (catalog number 9510, Cell Signaling Technology,

USA), Smad2/3 (catalog number 3102, Cell Signaling Technology), and glyceraldehyde 3-phosphate dehydrogenase (GAPDH) (Chemicon, Temecula, CA, USA). After rinsing with buffer, the membrane was stained with LI-COR Biosciences IRDye 800-labeled secondary antibodies (anti-goat/mouse/rabbit; catalog numbers 926-32214, 926-32212, and 926-32213, respectively; Rockland Immunochemicals) in the dark at room temperature. Positive signals were detected by the Odyssey infrared imaging system (LI-COR Biosciences) and quantitated with the ImageJ program (NIH). The ratio for the detected protein was normalized against GAPDH.

Real-time PCR

Total RNA was extracted from the LV by using TRIzol reagent (Invitrogen, Carlsbad, CA, USA) according to the manufacturer's protocol. The primer sequences used for detection of *TGF-β1*, *collagen I*, *collagen III*, *α-SMA*, *fibronectin*, *GAPDH*, *U6*, and *miR-29b* (*miR-29b-3p*) have been described previously,[27,28,31] and detailed sequences are listed in Table S1. Real-time PCR was conducted by using iQ SYBR Green Supermix with Opticon2 (Bio-Rad, Hercules, CA, USA) or the TaqMan microRNA assay (Applied Biosystems, Foster City, CA, USA), and detection was done using the CFX96 PCR system (Bio-Rad). The relative level of the specific gene detected was normalized against GAPDH or U6 (for miR-29b).

Cell culture and transient knockdown of Erbb4-IR

To isolate cardiomyocytes and CFs from neonatal mouse hearts, the differential adhesion method was employed. The heart tissue was dissociated with Blendzyme 4 (Roche, Indianapolis, IN, USA), and the cell suspension was neutralized with fetal bovine serum. After passing through a 40-μm sterile cell strainer, the cells were pre-plated for 1 h at 37°C with 5% CO_2 to separate the unattached cardiomyocytes from adhered CFs. Cardiomyocytes and CFs were identified by staining with α-SMA (Sigma) and vimentin (Santa Cruz Biotechnology) antibodies, respectively, and analyzed by flow cytometry. Cells with more than 95% positive staining of α-SMA or vimentin were identified as CFs, whereas those with negative staining were used as cardiomyocytes. Cells were stimulated with Ang II (1μM, Sigma) for 0, 3, 6, 12, and 24 h and examined for *Erbb4-IR* expression by real-time PCR.

To investigate the role of *Erbb4-IR in vitro*, CFs were transiently transfected with either an *Erbb4-IR* siRNA-pSuper.puro vector or an empty pSuper.puro vector using Lipofectamine 3000 (Invitrogen) in Opti-MEM reduced-serum medium (Invitrogen). After transfection, cells were stimulated with Ang II (1 μmol/L, Sigma) for 0, 3, 6, 12, and 24 h and examined for *Erbb4-IR* expression by real-time PCR. Three independent experiments were performed to ensure reliability of the results.

Statistical analysis

Results were expressed as mean ± standard error of the mean (SEM). Statistical analysis was performed using one-way analysis of variance (ANOVA) followed by a Newman-Keuls multiple-comparisons test using Prism 6.0 (GraphPad, San Diego, CA, USA).

DATA AVAILABILITY

The data presented in this study are available upon request from the corresponding authors.

SUPPLEMENTAL INFORMATION

Supplemental information can be found online at https://doi.org/10.1016/j.omtn.2023.06.017.

ACKNOWLEDGMENTS

This study was supported by the Lui Che Woo Institute of Innovative Medicine (CARE program), the Health and Medical Research Fund of Hong Kong (HMRF 07180516), the National Natural Science Foundation of China (82074378), the Luzhou-Southwest Medical University Joint Special Grant for High-level Talents (Hui-Yao Lan Team), the Science and Technology Department of Sichuan Province (2020YJ0442, 21ZDYF0348, 2022YFS0621, and 22ZDYF3797), The Research Fund of Southwest Medical University (2021ZKZD022 and 2021XJYJS02), the Luzhou-Southwest Medical University Science and Technology Strategic Cooperation Project (2021LZXNYD-P04), the Innovation Team Project of the Affiliated Traditional Chinese Medicine Hospital of Southwest Medical University (2022-CXTD-02 and 2022-CXTD-03), and the Guangdong-Hong Kong-Macao-Joint Labs Program from the Guangdong Science and Technology Department (2019B121205005).

AUTHOR CONTRIBUTIONS

J.-C.L. and L.D. performed animal studies and drafted the manuscript. Z.-J.H., X.-R.H., H.-L.W., and L.W. were responsible for collecting and analyzing data. S.-J.Y and H.-Y.L. were responsible for the design and supervision of the study and revised the manuscript.

DECLARATION OF INTERESTS

The authors declare no competing interests.

REFERENCES

1. Messerli, F.H., Rimoldi, S.F., and Bangalore, S. (2017). The Transition From Hypertension to Heart Failure: Contemporary Update. JACC. Heart Fail. *5*, 543–551.
2. Ahmed, S.H., Clark, L.L., Pennington, W.R., Webb, C.S., Bonnema, D.D., Leonardi, A.H., McClure, C.D., Spinale, F.G., and Zile, M.R. (2006). Matrix metalloproteinases/tissue inhibitors of metalloproteinases: relationship between changes in proteolytic determinants of matrix composition and structural, functional, and clinical manifestations of hypertensive heart disease. Circulation *113*, 2089–2096.
3. López, B., González, A., Querejeta, R., Larman, M., and Díez, J. (2020). Alterations in the pattern of collagen deposition may contribute to the deterioration of systolic function in hypertensive patients with heart failure. J. Am. Coll. Cardiol. *48*, 89–96.
4. Frangogiannis, N. (2020). Transforming growth factor-β in tissue fibrosis. J. Exp. Med. *217*, e20190103.
5. Meng, X.-M., Nikolic-Paterson, D.J., and Lan, H.Y. (2016). TGF-β: the master regulator of fibrosis. Nat. Rev. Nephrol. *12*, 325–338.
6. Rodríguez-Vita, J., Sánchez-López, E., Esteban, V., Rupérez, M., Egido, J., and Ruiz-Ortega, M. (2005). Angiotensin II activates the Smad pathway in vascular smooth muscle cells by a transforming growth factor-beta-independent mechanism. Circulation *111*, 2509–2517.
7. Wang, W., Huang, X.R., Canlas, E., Oka, K., Truong, L.D., Deng, C., Bhowmick, N.A., Ju, W., Bottinger, E.P., and Lan, H.Y. (2006). Essential role of Smad3 in angiotensin II-induced vascular fibrosis. Circ. Res. *98*, 1032–1039.

8. Yang, F., Chung, A.C.K., Huang, X.R., and Lan, H.Y. (2009). Angiotensin II induces connective tissue growth factor and collagen I expression via transforming growth factor-beta-dependent and -independent Smad pathways: the role of Smad3. Hypertension *54*, 877–884.

9. Yang, F., Huang, X.R., Chung, A.C.K., Hou, C.C., Lai, K.N., and Lan, H.Y. (2010). Essential role for Smad3 in angiotensin II-induced tubular epithelial-mesenchymal transition. J. Pathol. *221*, 390–401.

10. Huang, X.R., Chung, A.C.K., Yang, F., Yue, W., Deng, C., Lau, C.P., Tse, H.F., and Lan, H.Y. (2010). Smad3 mediates cardiac inflammation and fibrosis in angiotensin II-induced hypertensive cardiac remodeling. Hypertension *55*, 1165–1171.

11. Liu, Z., Huang, X.R., and Lan, H.Y. (2012). Smad3 mediates ANG II-induced hypertensive kidney disease in mice. Am. J. Physiol. Renal Physiol. *302*, F986–F997.

12. Meng, J., Qin, Y., Chen, J., Wei, L., Huang, X.R., Yu, X., and Lan, H.Y. (2020). Treatment of Hypertensive Heart Disease by Targeting Smad3 Signaling in Mice. Mol. Ther. Methods Clin. Dev. *18*, 791–802.

13. Yang, X., Letterio, J.J., Lechleider, R.J., Chen, L., Hayman, R., Gu, H., Roberts, A.B., and Deng, C. (1999). Targeted disruption of SMAD3 results in impaired mucosal immunity and diminished T cell responsiveness to TGF-beta. EMBO J. *18*, 1280–1291.

14. Greco, S., Salgado Somoza, A., Devaux, Y., and Martelli, F. (2018). Long Noncoding RNAs and Cardiac Disease. Antioxid. Redox Signal. *29*, 880–901.

15. Wang, Z., Zhang, X.J., Ji, Y.X., Zhang, P., Deng, K.Q., Gong, J., Ren, S., Wang, X., Chen, I., Wang, H., et al. (2016). The long noncoding RNA Chaer defines an epigenetic checkpoint in cardiac hypertrophy. Nat. Med. *22*, 1131–1139.

16. Greco, S., Zaccagnini, G., Perfetti, A., Fuschi, P., Valaperta, R., Voellenkle, C., Castelvecchio, S., Gaetano, C., Finato, N., Beltrami, A.P., et al. (2016). Long noncoding RNA dysregulation in ischemic heart failure. J. Transl. Med. *14*, 183.

17. Han, P., Li, W., Lin, C.H., Yang, J., Shang, C., Nuernberg, S.T., Jin, K.K., Xu, W., Lin, C.Y., Lin, C.J., et al. (2014). A long noncoding RNA protects the heart from pathological hypertrophy. Nature *514*, 102–106.

18. Viereck, J., Kumarswamy, R., Foinquinos, A., Xiao, K., Avramopoulos, P., Kunz, M., Dittrich, M., Maetzig, T., Zimmer, K., Remke, J., et al. (2016). Long noncoding RNA Chast promotes cardiac remodeling. Sci. Transl. Med. *8*, 326ra22.

19. Zhou, Q., Chung, A.C.K., Huang, X.R., Dong, Y., Yu, X., and Lan, H.Y. (2014). Identification of novel long noncoding RNAs associated with TGF-β/Smad3-mediated renal inflammation and fibrosis by RNA sequencing. Am. J. Pathol. *184*, 409–417.

20. Zhou, Q., Huang, X.R., Yu, J., Yu, X., Lan, H.Y., and Zhou, Q. (2015). Long Noncoding RNA Arid2-IR Is a Novel Therapeutic Target for Renal Inflammation. Mol. Ther. *23*, 1034–1043.

21. Feng, M., Tang, P.M.K., Huang, X.R., Sun, S.F., You, Y.K., Xiao, J., Lv, L.L., Xu, A.P., and Lan, H.Y. (2018). TGF-β Mediates Renal Fibrosis via the Smad3-Erbb4-IR Long Noncoding RNA Axis. Mol. Ther. *26*, 148–161.

22. Sun, S.F., Tang, P.M.K., Feng, M., Xiao, J., Huang, X.R., Li, P., Ma, R.C.W., and Lan, H.Y. (2018). Novel lncRNA Erbb4-IR Promotes Diabetic Kidney Injury in Mice by Targeting miR-29b. Diabetes *67*, 731–744.

23. Meng, X.M., Huang, X.R., Chung, A.C.K., Qin, W., Shao, X., Igarashi, P., Ju, W., Bottinger, E.P., and Lan, H.Y. (2010). Smad2 protects against TGF-beta/Smad3-mediated renal fibrosis. J. Am. Soc. Nephrol. *21*, 1477–1487.

24. Khalil, H., Kanisicak, O., Prasad, V., Correll, R.N., Fu, X., Schips, T., Vagnozzi, R.J., Liu, R., Huynh, T., Lee, S.J., et al. (2017). Fibroblast-specific TGF-β-Smad2/3 signaling underlies cardiac fibrosis. J. Clin. Invest. *127*, 3770–3783.

25. Kong, P., Shinde, A.V., Su, Y., Russo, I., Chen, B., Saxena, A., Conway, S.J., Graff, J.M., and Frangogiannis, N.G. (2018). Opposing Actions of Fibroblast and Cardiomyocyte Smad3 Signaling in the Infarcted Myocardium. Circulation *137*, 707–724.

26. Huang, S., Chen, B., Su, Y., Alex, L., Humeres, C., Shinde, A.V., Conway, S.J., and Frangogiannis, N.G. (2019). Distinct roles of myofibroblast-specific Smad2 and Smad3 signaling in repair and remodeling of the infarcted heart. J. Mol. Cell. Cardiol. *132*, 84–97.

27. Wei, L.H., Huang, X.R., Zhang, Y., Li, Y.Q., Chen, H.Y., Heuchel, R., Yan, B.P., Yu, C.M., and Lan, H.Y. (2013). Deficiency of Smad7 enhances cardiac remodeling induced by angiotensin II infusion in a mouse model of hypertension. PLoS One *8*, e70195.

28. Wei, L.H., Huang, X.R., Zhang, Y., Li, Y.Q., Chen, H.Y., Yan, B.P., Yu, C.M., and Lan, H.Y. (2013). Smad7 inhibits angiotensin II-induced hypertensive cardiac remodelling. Cardiovasc. Res. *99*, 665–673.

29. van Rooij, E., Sutherland, L.B., Thatcher, J.E., DiMaio, J.M., Naseem, R.H., Marshall, W.S., Hill, J.A., and Olson, E.N. (2008). Dysregulation of microRNAs after myocardial infarction reveals a role of miR-29 in cardiac fibrosis. Proc. Natl. Acad. Sci. USA *105*, 13027–13032.

30. Li, C., Wang, N., Rao, P., Wang, L., Lu, D., and Sun, L. (2021). Role of the microRNA-29 family in myocardial fibrosis. J. Physiol. Biochem. *77*, 365–376.

31. Zhang, Y., Huang, X.R., Wei, L.H., Chung, A.C., Yu, C.M., and Lan, H.Y. (2014). miR-29b as a therapeutic agent for angiotensin II-induced cardiac fibrosis by targeting TGF-β/Smad3 signaling. Mol. Ther. *22*, 974–985.

32. Qin, W., Chung, A.C.K., Huang, X.R., Meng, X.M., Hui, D.S.C., Yu, C.M., Sung, J.J.Y., and Lan, H.Y. (2011). TGF-β/Smad3 signaling promotes renal fibrosis by inhibiting miR-29. J. Am. Soc. Nephrol. *22*, 1462–1474.

33. Xiao, J., Meng, X.M., Huang, X.R., Chung, A.C., Feng, Y.L., Hui, D.S., Yu, C.M., Sung, J.J., and Lan, H.Y. (2012). miR-29 inhibits bleomycin-induced pulmonary fibrosis in mice. Mol. Ther. *20*, 1251–1260.

34. Lan, H.Y., Mu, W., Tomita, N., Huang, X.R., Li, J.H., Zhu, H.J., Morishita, R., and Johnson, R.J. (2003). Inhibition of renal fibrosis by gene transfer of inducible Smad7 using ultrasound-microbubble system in rat UUO model. J. Am. Soc. Nephrol. *14*, 1535–1548.

OMTN, Volume *33*

Supplemental information

Angiotensin II mediates

hypertensive cardiac fibrosis

via an Erbb4-IR-dependent mechanism

Jian-Chun Li, Jian Jia, Li Dong, Zhong-Jing Hu, Xiao-Ru Huang, Hong-Lian Wang, Li Wang, Si-Jin Yang, and Hui-Yao Lan

Table S1. List of PCR primers.

Primers	Forward 5'-3'	Reverse 5'-3'
Erbb4-IR	AACTCGCCACAGAAATCCAC	ACAACCCCAAACAAGCTGTC
GAPDH	TGCTGAGTATGTCGTGGAGTCTA	AGTGGGAGTTGCTGTTGAAATC
TGF-β1	CAACAATTCCTGGCGTTACCTTGG	GAAAGCCCTGTATTCCGTCTCCTT
collagen I	GAGCGGAGAGTACTGGATCG	TACTCGAACGGGAATCCATC
collagen III	CTGGACCAAAAGGTGATGCTG	TGCCAGGGAATCCTCGATGTC
α-SMA	ACTGGGACGACATGGAAAAG	CATCTCCAGAGTCCAGCACA
fibronectin	ACACGGTTTCCCATTACGCCAT	AATGACCACTGCCAAAGCCCAA
Smad7	GTGCTATGTCGCTCTGGACTTTGA	ATGAAAGATGGCTGGAAGAGGGTC
mCherry	TTCATGTACGGCTCCAAGGC	TGTAGATGAACTCGCCGTCC
miR-29b	ATCTTTGTATCTAGCACCATT	AATGGTGCTAGATACAAAGAT
U6(universal)	GTGCTCGCTTCGGCAGCACATATACTAAAATTGGAACGATACAGAGAAGATTAGCATGGCCCCTGCGCAAGGATGACACGCAAATTCGTGAAGCGTTCCATATTTT	

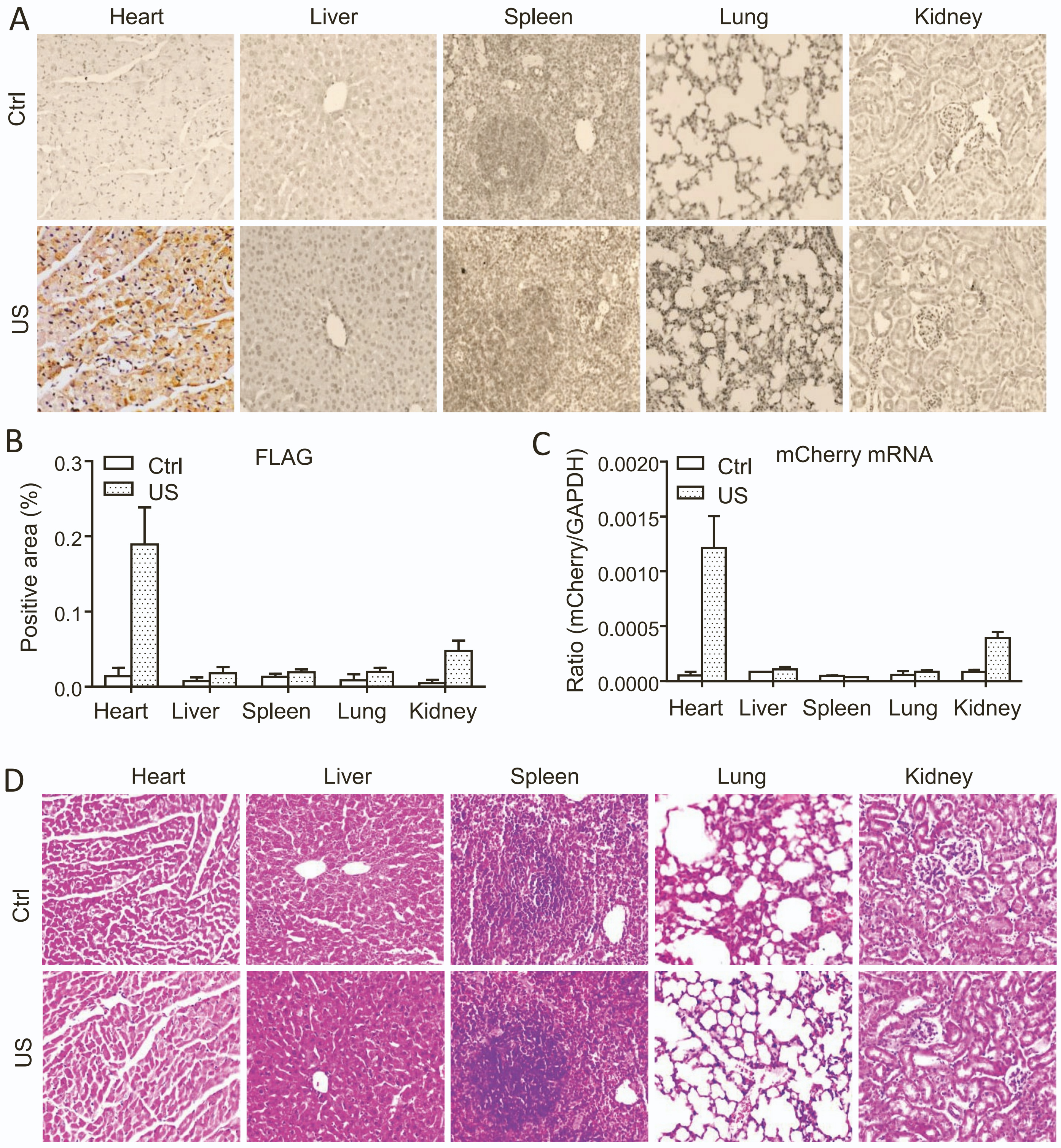

Figure S1. Ultrasound-microbubble mediated cardiac-specific FLAG-mCherry (reporter gene) expression without detectable organ histological injury. (**A,B**) Immunohistochemistry and quantitative analysis for FLAG-mCherry protein expression. (**C**) Real-time PCR for mCherry mRNA expression. (**D**) PAS-staining. Data represent as the mean±SD for groups of 3 mice.
